# Acupuncture for chemotherapy-associated insomnia in breast cancer patients: an assessor-participant blinded, randomized, sham-controlled trial

**DOI:** 10.1186/s13058-023-01645-0

**Published:** 2023-04-26

**Authors:** Jialing Zhang, Zongshi Qin, Tsz Him So, Tien Yee Chang, Sichang Yang, Haiyong Chen, Wing Fai Yeung, Ka Fai Chung, Pui Yan Chan, Yong Huang, Shifen Xu, Chun Yuan Chiang, Lixing Lao, Zhang-Jin Zhang

**Affiliations:** 1 School of Chinese Medicine, LKS Faculty of Medicine, The University of Hong Kong, 10 Sassoon Road, Pokfulam, Hong Kong China; 2 School of Chinese Medicine, Hong Kong Baptist University, Kowloon Tong, Hong Kong China; 3 Department of Clinical Oncology, LKS Faculty of Medicine, The University of Hong Kong, Pokfulam, Hong Kong China; 4 Comprehensive Oncology Centre, Hong Kong Sanatorium & Hospital, Happy Valley, Hong Kong China; 5 School of Nursing, The Hong Kong Polytechnic University, Hung Hom, Hong Kong China; 6 Department of Psychiatry, The University of Hong Kong, Pokfulam, Hong Kong China; 7 School of Traditional Chinese Medicine, Southern Medical University, Guangzhou, 510515 Guangdong China; 8 Shanghai Municipal Hospital of Traditional Chinese Medicine, Shanghai University of Traditional Chinese Medicine, Shanghai, 200071 China; 9 Hong Kong Institute of Cell & Molecular Medicine and Digital Centre of State-Key-Laboratory on Quality Appraisal of TCM, Hong Kong, China; 10 Virginia University of Integrative Medicine, Fairfax, VA 22031 USA; 11 Department of Chinese Medicine, The University of Hong Kong Shenzhen Hospital (HKU-SZH), Shenzhen, 518053 Guangdong China

**Keywords:** Chemotherapy-associated insomnia, Breast cancer, Acupuncture, Cessation rate of sleeping medications

## Abstract

**Background:**

Insomnia is a highly prevalent symptom occurred during and post-chemotherapy. Acupuncture may have beneficial effects in the management of chemotherapy-associated insomnia. This study was conducted to determine the efficacy and safety of acupuncture in improving chemotherapy-associated insomnia in breast cancer patients.

**Methods:**

This assessor-participant blinded, randomized, sham-controlled trial was conducted from November 2019 to January 2022 (follow-up completed July 2022). Participants were referred by oncologists from two Hong Kong hospitals. Assessments and interventions were conducted at the outpatient clinic of School of Chinese Medicine, the University of Hong Kong. The 138 breast cancer patients with chemotherapy-associated insomnia were randomly assigned to receive either 15 sessions of active acupuncture regimen by combining needling into body acupoints and acupressure on auricular acupoints or sham acupuncture control (69 each) for 18 weeks, followed by 24 weeks of follow-up. The primary outcome was measured using Insomnia Severity Index (ISI). Secondary outcomes included the Pittsburgh Sleep Quality Index, Actiwatch and sleep diary for sleep parameters, depression and anxiety, fatigue and pain, and quality of life.

**Results:**

There were 87.7% (121/138) participants who completed the primary endpoint (week-6). The active acupuncture regimen was not superior to the sham control in reducing ISI score from baseline to 6 weeks (mean difference: − 0.4, 95% CI − 1.8–1.1; *P* = 0.609), but produced short-term treatment and long-term follow-up better outcomes in improving sleep onset latency, total sleep time, sleep efficiency, anxiety, depression, and quality of life. Participants of the active acupuncture group had a pronouncedly higher cessation rate of sleeping medications than the sham control (56.5% vs. 14.3%, *P* = 0.011). All treatment-related adverse events were mild. No participants discontinued treatments due to adverse events.

**Conclusion:**

The active acupuncture regimen could be considered as an effective option for the management of chemotherapy-associated insomnia. It also could serve as a tapering approach to reduce and even replace the use of sleeping medications in breast cancer patients.

*Trial registration* Clinicaltrials.gov: NCT04144309. Registered 30 October 2019.

**Supplementary Information:**

The online version contains supplementary material available at 10.1186/s13058-023-01645-0.

## Background

Breast cancer patients are at significantly greater risk of developing insomnia than those with other malignancies and the general population [1, 2]. Individuals under chemotherapy experienced higher incidence and more severe levels of insomnia than those who received other cancer treatments such as surgery and radiotherapy [2–4]. More than 1/4 of breast cancer patients developed new-onset insomnia during chemotherapy, and more likely to persist after the completion of cancer treatments than those without chemotherapy [5, 6]. The development of chemotherapy-associated insomnia in breast cancer patients is associated with multiple factors which may trigger and even aggravate insomnia, mainly including psychological stress due to cancer diagnosis and treatments, direct effects of medications and chemotherapy, reduction in physical activity, disruption in circadian, and somatic-cognitive-cortical hyperarousal [7–10]. The presence of insomnia not only increases risks of psychiatric and physical comorbidities [11, 12], but also reduces patients’ willingness to complete cancer treatments and decreases quality of life. Although pharmacological therapy is the mainstay of the management of insomnia in cancer patients, it is generally recommended for short-term use, as sedative-hypnotic medications often cause undesirable side effects, particularly dependence and tolerance [13]. Cognitive behavioral therapy may be efficacious for improving sleep quality in breast cancer women [14], but intensive labour requirements and high treatment costs have limited the access to this non-pharmacological therapy [15, 16].

Over the past two decades, acupuncture has gained increasing popularity in the treatment of insomnia, cancer and cancer-related symptoms [17–19]. Numerous clinical practice guidelines have recommended acupuncture as an effective option for chemotherapy-induced side effects [17, 20]. There have been a large number of clinical trials that demonstrated promising efficacy of acupuncture for various types of insomnia [21, 22]. However, the effectiveness of acupuncture for chemotherapy-associated insomnia remains for further evaluation. Our recent pilot-controlled trial has shown the potential benefits of the combination of needling into body acupoints and auricular acupressure in 30 breast cancer patients who underwent chemotherapy and experienced insomnia [23]. We therefore hypothesized that the combination acupuncture regimen could produce better outcomes than sham regimen in improving chemotherapy-associated insomnia. To test this hypothesis, an assessor-participant blinded, randomized, sham-controlled trial was designed to compare the efficacy and safety of the two regimens in breast cancer patients with chemotherapy-associated insomnia.

## Methods

### Design and settings

This was an assessor-participant-data analyst blinded, randomized, sham-controlled trial. The trial was approved by Institutional Review Board of the University of Hong Kong (HKU)/Hospital Authority Hong Kong (HK) West Cluster (UW 19-045) and Research Ethics Committee of HK Sanatorium & Hospital (REC-2019-14). The trial was registered in www.clinicaltrials.gov (NCT04144309). The study was conducted in accordance with Standards for Reporting Interventions in Clinical Trials of Acupuncture and Consolidated Standards of Reporting Trials statement [24, 25].

Recruitment and treatment occurred between November 2019 and January 2022; follow-up assessments completed in July 2022. Participants were referred by oncologists from HK Queen Mary Hospital and HK Sanatorium & Hospital. Multiple promotions were carried out to facilitate recruitment, including advertising on newspapers and Facebook. Participants gave voluntary, written, informed consent after study procedures were fully explained. Assessments and interventions were conducted at the outpatient clinic of School of Chinese Medicine, HKU.

### Participants

Patients were eligible if they were: (i) females aged 18–75; (ii) had a diagnosis of stage I–IV breast cancer; (iii) underwent or had completed chemotherapy no more than 6 months; (iv) insomnia occurred at least 3 nights/week and lasted at least one month, with the fulfilment of the diagnostic criteria for brief insomnia disorder of the Diagnostic and Statistical Manual of Mental Disorders (5th Edition); and (v) the severity of insomnia reached at least 10 points of Insomnia Severity Index (ISI) over the past 2 weeks.

Patients were excluded if they had: (i) other sleep disorders (e.g., obstructive sleep apnoea), irregular sleep pattern, or shift work; (ii) severe hearing, visual or language defects; (iii) severe hematological dysfunction (e.g., haemoglobin < 8 g/dL, platelet count < 60,000/μL, absolute neutrophil count < 1000/μL); (iv) pacemakers or other electronic implants that might interfere with electroacupuncture; (v) had acupuncture treatment in the past 3 months; or (vi) participated in other clinical trials in the past 3 months.

### Randomization

Permuted block randomization (block sizes of 2/4/6) was used. After baseline assessments, participants were randomly assigned to two groups at a ratio of 1:1 according to the randomization sequence. The randomization sequence was generated by an independent biostatistician using Microsoft Excel prior to study initiation. Individual randomization code was sealed in sequentially numbered opaque envelopes and opened by acupuncturist after participants completed baseline assessments.

### Blinding and allocation concealment

Investigators and research staffs who performed screening, assessments, and data entry/re-entry/analysis were blinded to group allocation. Participants were informed that they would have same chance of allocating to either group and would be blinded to allocation. Treatment was delivered individually in a separate room to avoid communications among participants about treatment experience. Eye mask was used to block participant’s vision during treatment so that she was unaware of acupuncture procedure. Acupuncturists were instructed not to acquire participants’ information except group allocation. Interactions between acupuncturists and participants were kept to a minimum to avoid accidental disclosure of group allocation.

### Intervention

Participants continued routine care and symptom management during study. Acupuncture treatment was carried out by registered Chinese Medicine Practitioners (CMPs) had at least 5-year clinical practice experience and completed training workshop prior to study initiation. The training workshop included introduction of treatment protocol, standard procedures of active and sham acupuncture, and conversation skills with participants. The treatment protocol [26] was developed based on neuroanatomic mechanisms [27, 28], CMPs’ experience and previous clinical trials [21, 29]. Participants received a total of 15 sessions of treatments, with 12 sessions given twice weekly as intensive treatment over the first 6 consecutive weeks, 3 sessions given once every 4 weeks from 7 to 18 weeks to maintain treatment effects, and follow-up from 19 to 42 weeks.

#### Active acupuncture regimen

Active acupuncture regimen consisted of electroacupuncture on body acupoints and auricular acupressure. Six fixed acupoints (EX-HN1, GV20, GV24, PC6, KI3 and SP6) were utilized for treating insomnia [30–33]. Four additional acupoints were selected based on comorbid symptoms (Table 1). Locations/therapeutic effects of acupoints are summarized in Additional file 1: eTable S1A. Details procedures are listed in Additional file 1: eTable S1B. Auricular acupressure was conducted by embedding black, hard Vaccaria seeds on surface of bilateral 3 auricular points (Heart, Shenmen and Sympathetic).

**Table 1:** Recommendation of additional acupoints based on comorbid symptoms

Comorbid symptoms	Recommended acupoints	
Dizziness	EX-HN3 (Yintang)	ST36 (Zusanli)
Headache	EX-HN3 (Yintang)	LI4 (Hegu)
Fatigue	ST36 (Zusanli)	CV4 (Guanyuan)
Hot flushes	KI3 (Taixi)	CV4 (Guanyuan)
depression/anxiety	EX-HN3 (Yintang)	LR3 (Taichong)
Nausea/vomiting	ST36 (Zusanli)	LI4 (Hegu)
Loss of appetite	ST36 (Zusanli)	LI4 (Hegu)
Diarrhea/constipation	ST25 (Tianshu)	ST36 (Zusanli)

#### Sham acupuncture regimen

Sham points that are located at 1–2 cm adjacent to meridian-based acupoints were used for sham electroacupuncture [34]. Streitberger’s non-invasive retractable needles were compressed via guiding tubes onto point skin [35]. For sham auricular acupressure, 3 sham points in helix region (HX7, HX8, HX9) that are located remotely from inner ear area and their effects are not indicated for insomnia were selected [36]. Soft stem piths of Medulla Junci were cut and dyed in black were used to mimic auricular acupressure [37].

#### Concomitant use of psychotropic medications

Psychotropic medications were allowed to be prescribed at the discretion of psychiatrists and general physicians during study. The proportion of participants who were prescribed with sleeping medications (sedatives, hypnotics and anxiolytics), dosage and weekly frequency of use were recorded. The dosage used was converted as diazepam equivalent dosage. Four mean dosages were obtained by averaging across a 2 week duration [38]: prior to entry, post-treatment 6 weeks and 18 weeks, and prior to the end of follow-up. Cessation rate of sleeping medications was calculated.

### Assessments

#### Primary outcome

Details of outcomes have been reported previously [26]. The primary outcome was defined as the change in ISI score at 6 weeks of treatment from baseline. ISI is a 7-item self-rating questionnaire that has good internal consistency and construct validity in differentiating insomnia severity in cancer patients [39]. ISI score ranges from 0 to 28. The higher score represents more severe insomnia, with 10 points indicative of subthreshold level of insomnia [40, 41].

#### Secondary outcomes

Secondary outcomes included sleeping measures, depression and anxiety, fatigue and pain, and quality of life. Actiwatch [42] (Spectrum Plus, Philips Respironics, USA) and Sleep Diary [43] were used to record sleep onset latency (SOL), wake time after sleep onset (WASO), total sleep time (TST), and sleep efficiency (SE). Participants were required to record for 2 sessions of 7 consecutive nights starting at baseline and starting after 6 week of treatment. Pittsburgh Sleep Quality Index (PSQI) was a 19-item questionnaire used to assess sleep dysfunction, with higher score indicating poorer sleep quality [44, 45].

The severity of depression/anxiety was measured using Hospital Anxiety and Depression Scale (HADS) which is a 14-item questionnaire with two subscales to evaluate the severity of depressive and anxiety symptoms [46, 47]. Fatigue and pain symptoms were measured using Brief Fatigue Inventory (BFI) and Brief Pain Inventory-Short Form (BPI-SF), respectively. BFI is a 9-item self-rating scale designed to evaluate the severity and impact of cancer-related fatigue on daily functioning, with higher score corresponding to greater level of fatigue [48]. BPI-SF is a self-administered questionnaire developed to evaluate pain severity and pain interference on daily function over the past 24 h [49, 50]. Quality of life was measured using Functional Assessment of Cancer Therapy-Breast Cancer (FACT-B). FACT-B is a 37-item self-reported instrument devised to assess multidimensional health-related quality of life in breast cancer patients [51], with higher score indicating better quality of life [52]. Questionnaires were completed at baseline, week-3, 6, 10, 14, 18, 30 and 42.

#### Adverse events

Adverse events (AEs) were recorded during study. Whether an AE related to treatment was determined by acupuncturists. Serious AEs were immediately reported to principal investigator and Ethics Committee within 24 h of the occurrence.

### Credibility, expectancy, adherence, and successiveness of blinding

The credibility of treatment was assessed using the 4-item, 6-point Credibility Rating Scale [53]. Expectancy for acupuncture outcomes was assessed using the 4-item 5-point Acupuncture Expectancy Scale [54]. Greater scores of both scales represent stronger confidence and higher expectancy to treatment. Adherence was assessed by counting the number of completed treatment sessions. Participants were asked to guess which type of acupuncture they had received after the third treatment [55]. James’ and Bang’s blinding index were calculated to evaluate successiveness of blinding [56].

### Data security and monitoring

All data were secured in compliance with HK Personal Data (Privacy) Ordinance (CAP 486). A data and safety monitoring board (DSMB) was established [57] and comprised of an independent biostatistician, an oncologist and a psychiatrist. They were not involved in the conduct of the trial. DSMB meetings were held prior to the initiation of recruitment and after the final analysis. To prevent loss of follow-up, text messages were sent or phone calls were made to individual participants a day before scheduled visits. The research team held weekly meetings to troubleshoot issues pertaining to patient recruitment and retention.

### Statistical analysis

#### Estimation of sample size

There was no study has previously been conducted in the female with breast cancer experiencing chemotherapy-associated insomnia. The sample size was estimated based on anticipated changes of ISI score. Based on previous trials among non-cancer population with primary insomnia [21, 58], in which the reduction of ISI score was between 2.3 and 5.0, with median value of 2.5 and pooled standard deviation of 4.7. The study was assumed to detect a 2.5 difference between groups in reduced ISI score, with a 95% level of significance and an 80% power. A total of 138 subjects (69/group) were needed in the consideration of 20% dropout rate.

#### Data analysis

The efficacy was analyzed in the intention-to-treat population, defined as participants who completed baseline assessments. For missing data, the multiple imputation method was used under the missing-at-random (MAR) assumption. The sensitivity of MAR assumption of missing data was tested with a pattern-mixture model [59], in which the robustness of the analyzed results of primary outcome was examined. The primary outcome and continuous secondary outcomes were compared using a mixed-effect model adjusted with baseline values, with time, group, and interaction between time and group as the fixed effects, and individual subject as the random effect.

Categorical variables, including categorical baseline variables, cessation rate of sleeping medications and incidence of AEs, were analyzed using Chi-square or Fisher’s exact tests. Unpaired *t*-test or Mann–Whitney U test was used to compare continuous variables between groups, including baseline variables, changes in Actiwatch-/diary-recorded variables, credibility score and medication dosage. Statistical analyses were performed with SPSS version 26.0 (IBM Corporation, Armonk, NY) and SAS version 9.4 (SAS Inc.). Hypothesis testing was carried out at 5% (2-sided) significance level.

## Results

### Participant characteristics

**Fig. 1:**
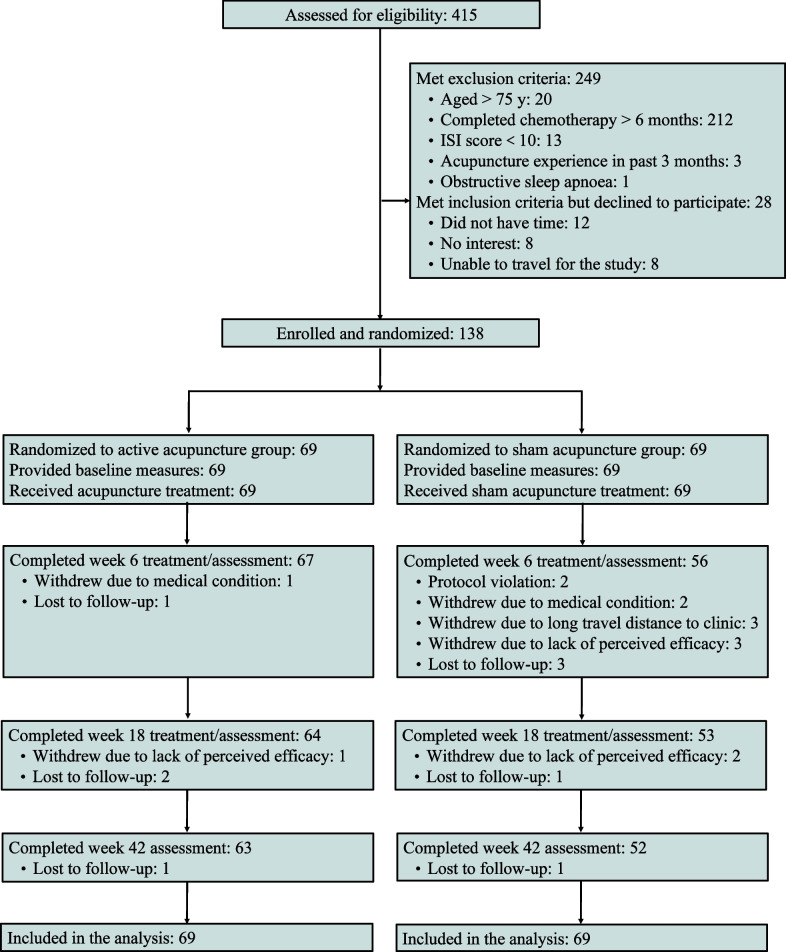
Flow diagram of progress of recruitment, treatment and follow-up

Of 415 patients screened, 166 eligible, but 28 declined to participate (Fig. 1). The remaining 138 were randomly assigned to the active and sham acupuncture groups (69/group). The proportion of participants who completed at least 12 treatments was 97.1% (67/69) in the active acupuncture group and 81.2% (56/69) in the sham control group. The discontinuation rate was 10.9% (15/138) over the first 6 weeks and 16.7% (23/138) over the whole trial. The overall discontinuation rate of the active acupuncture was pronouncedly lower than that of the sham control [8.7% (6/69) vs. 24.6% (17/69), *P* = 0.012].

Demographic and baseline variables were similar between groups. All participants were Asian women with mean (SD) age of 52.2 (9.0) years. The mean ISI score was 17.1 (4.4) points, with the duration of insomnia of 10.0 (6.7) months. There were 32.6% (45/138) participants who were taking sleeping medications at entry (Table 2).

**Table 2:** Participants characteristics

Characteristic	Active acupuncture (*n* = 69)	Sham acupuncture (*n* = 69)	Total (*n* = 138)	*P* value*
Age, years	51.7 ± 9.6	52.7 ± 8.3	52.2 ± 9.0	0.540
Body mass index, kg/m^2^	22.8 ± 2.9	24.0 ± 16.9	23.4 ± 12.1	0.581
Marital status				0.586
Married or living with partner	48 (69.6)	45 (65.2)	93 (67.4)	
Single, separated, divorced, or widowed	21 (30.4)	24 (34.8)	45 (32.6)	
Educational attainment				0.959
Primary or below	7 (10.1)	8 (11.6)	15 (10.9)	
Secondary	33 (47.8)	33 (47.8)	66 (47.8)	
Post-secondary or above	29 (42.0)	28 (40.6)	57 (41.3)	
Household monthly income (HK$)				0.111
< 20,000	36 (52.2)	39 (56.5)	75 (54.3)	
20,000–50,000	23 (33.3)	13 (18.8)	36 (26.1)	
> 50,000	10 (14.5)	15 (21.7)	25 (18.1)	
No answer	0 (0.0)	2 (2.9)	2 (1.4)	
Occupation				0.384
Professional and associate professional	22 (31.9)	20 (29.0)	42 (30.4)	
Skilled and semi-skilled worker	3 (4.3)	3 (4.3)	6 (4.3)	
Unskilled worker	19 (27.5)	12 (17.4)	31 (22.5)	
Retired/unemployed/housework	25 (36.2)	34 (49.3)	59 (42.8)	
Menopausal status at entry				0.390
Premenopausal	4 (5.8)	1 (1.4)	5 (3.6)	
Perimenopausal	13 (18.8)	13 (18.8)	26 (18.8)	
Postmenopausal	52 (75.4)	55 (79.7)	107 (77.5)	
Breast cancer stage				0.479
I	13 (18.8)	19 (27.5)	32 (23.2)	
II	29 (42.0)	26 (37.7)	55 (39.9)	
III	15 (21.7)	17 (24.6)	32 (23.2)	
IV	10 (14.5)	7 (10.1)	17 (12.3)	
No answer	2 (2.9)	0 (0.0)	2 (1.4)	
Prior surgery	61 (88.4)	64 (92.8)	125 (90.6)	0.382
Prior radiotherapy	37 (53.6)	40 (58.0)	77 (55.8)	0.607
Prior hormonal therapy	34 (49.3)	38 (55.1)	72 (52.2)	0.495
Adjuvant Chemotherapy	51 (73.9)	57 (82.6)	108 (78.3)	0.417
Under or post-chemotherapy at entry				0.687
Under	15 (21.7)	17 (24.6)	32 (23.2)	
Post	54 (78.3)	52 (75.4)	72 (76.8)	
Chemotherapy regimens				0.583
AC/TC	14 (20.3)	20 (29.0)	34 (24.6)	
TAC	6 (8.7)	10 (14.5)	16 (11.6)	
AC/EC + T/P	13 (18.8)	9 (13.0)	22 (15.9)	
FEC + T	8 (11.6)	5 (7.2)	13 (9.4)	
Carboplatin-containing	17 (24.6)	14 (20.3)	31 (22.5)	
Others	11 (15.9)	11 (15.9)	22 (15.9)	
Insomnia mean duration, months^a^	9.0 (3.0, 13.5)	10.0 (5.5, 15.0)	9.0 (4.8, 14.0)	0.141
Sleep aids, prior 2 weeks				
Sleep medications	26 (37.7)	19 (27.5)	45 (32.6)	0.204
Chinese herbal medicine	16 (23.2)	24 (34.8)	40 (29.0)	0.133
Prior acupuncture	50 (72.5)	46 (66.7)	96 (69.6)	0.459
ISI	17.4 ± 4.3	16.7 ± 4.5	17.1 ± 4.4	0.358
PSQI	13.6 ± 3.4	12.7 ± 3.3	13.1 ± 3.4	0.100
Actiwatch				
SOL, min	14.2 ± 14.5	14.2 ± 12.3	14.2 ± 13.4	0.976
WASO, min	112.6 ± 40.2	112.5 ± 38.4	112.5 ± 39.2	0.991
TST, min	359.1 ± 51.1	357.7 ± 51.3	358.4 ± 51.0	0.878
SE, %	72.8 ± 7.9	72.9 ± 7.5	72.8 ± 7.7	0.938
Sleep diary				
SOL, min	51.5 ± 38.6	47.8 ± 35.7	49.6 ± 37.1	0.558
WASO, min	58.0 ± 44.0	56.2 ± 47.1	57.1 ± 45.4	0.820
TST, min	319.4 ± 85.2	331.1 ± 96.8	325.3 ± 91.1	0.453
SE, %	64.8 ± 15.4	68.2 ± 17.9	66.5 ± 16.8	0.231
HADS				
Anxiety	9.2 ± 3.9	8.6 ± 3.2	8.9 ± 3.6	0.316
Depression	8.4 ± 4.1	8.1 ± 3.8	8.3 ± 3.9	0.682
BFI	5.6 ± 2.1	5.6 ± 2.1	5.6 ± 2.1	0.939
BPI-SF				
Pain severity	3.4 ± 2.9	4.3 ± 2.4	3.9 ± 2.7	0.054
Pain interference	3.3 ± 3.1	3.9 ± 2.7	3.6 ± 2.9	0.203
FACT-B	80.3 ± 21.3	82.5 ± 19.7	81.4 ± 20.4	0.536
AES	14.8 ± 3.4	14.8 ± 3.3	14.8 ± 3.3	0.980

Data are presented as mean ± standard deviation, number (%), or median (interquartile range)

*AC/TC* Adriamycin and cyclophosphamide, or taxotere and cyclophosphamide; *AC/EC* Adriamycin and cyclophosphamide, or epirubicin and cyclophosphamide; *BFI* Brief fatigue inventory; *BPI-SF* Brief pain inventory-short form; *FEC* + *T* Fluorouracil, epirubicin and cyclophosphamide, plus taxotere; *ISI* Insomnia severity index; *PSQI* Pittsburgh sleep quality index; *SOL* Sleep onset latency; *TAC* Taxotere, adriamycin, and cyclophosphamide; *T/P* Taxotere or paclitaxel; *WASO* Wake after sleep onset; *TST* Total sleep time; *SE* Sleep efficiency; *HADS* Hospital anxiety and depression scale; *FACT-B* Functional assessment of cancer therapy-breast cancer; *AES* Acupuncture expectancy scale

^a^Duration of insomnia was reported by the participant and verified by assessors before enrollment

*Comparison between acupuncture group and sham acupuncture group by $\chi^{2}$ or Fisher’s exact test, unpaired *t*-test, or Mann–Whitney U test

### Cessation rate and sleeping medications used

**Fig. 2:**
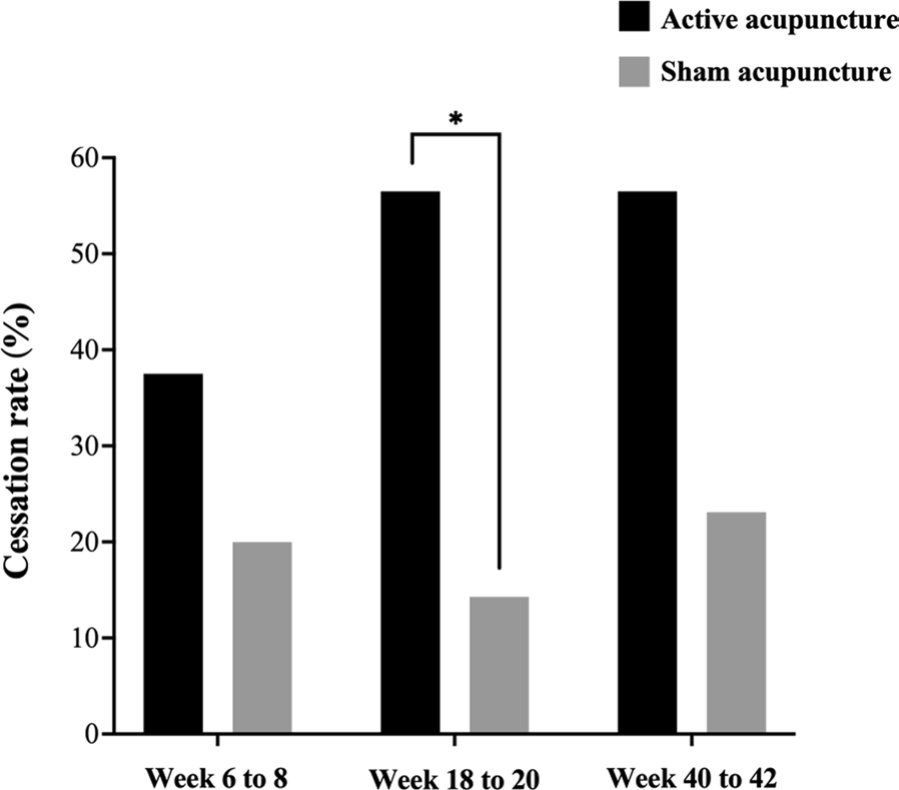
Cessation rate over time. *P < 0.05, comparison between groups by $\chi^{2}$ test

The active acupuncture group had a higher cessation rate of sleeping medication during 18–20 weeks than the sham control (56.5% vs. 14.3%, *P* = 0.011). Dosage of sleeping medications and weekly frequency of use were not significantly different between groups (Fig. 2, Additional file 1: eTable S2A). Characteristics of participants use sedatives, hypnotics, anxiolytics in two groups were similar (Fig. 2, Additional file 1: eTable S2B).

### Primary outcome

**Fig. 3:**
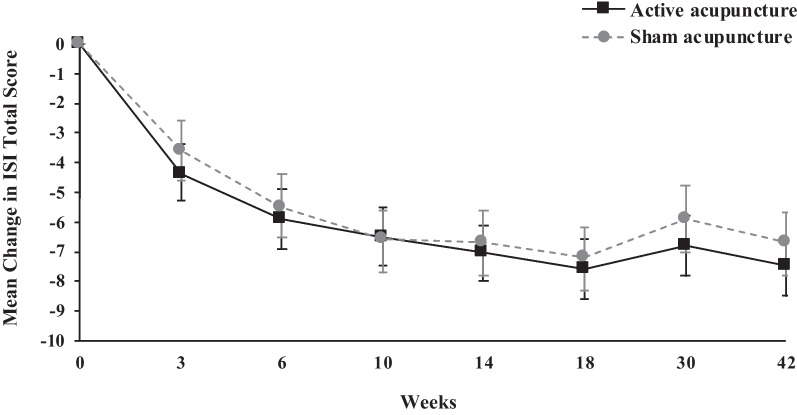
Mean change of ISI total score over time. Error bar represents 95% confidence interval

The ISI-measured severity of insomnia strikingly reduced from baseline over the 42 week course in two groups (all *Ps* < 0.001), but the magnitude of ISI score reduction was not significantly different between groups at any measurement points, including at 6 weeks with a 0.4 difference between the two groups (95% CI − 1.8–1.1; *P* = 0.609 (Table 3, Fig. 3). The tipping-point sensitivity analysis of shift ISI score showed that the non-differences were not reversed under missing at random with ISI score shifted from the minimum of 7 to the maximum of 28 (Additional file 1: eTable S3). Under the adjustment for baseline expectancy for treatment outcomes, the results did not show deviations (Additional file 1: eTable S4).

**Table 3:** Changes in outcomes from baseline by groups of sham-controlled study

Outcomes	Change from baseline		Between-group difference	*P* value^a^
	Active acupuncture (*n* = 69)	Sham acupuncture (*n* = 69)	Active acupuncture versus Sham acupuncture	
*Primary outcome*				
ISI†				
Week-3	− 4.4 (− 5.4 to − 3.5)^#^	− 3.6 (− 4.6 to − 2.6)^#^	− 0.8 (− 2.2 to 0.6)	0.265
Week-6	− 5.9 (− 6.9 to − 4.9)^#^	− 5.5 (− 6.6 to − 4.5)^#^	− 0.4 (− 1.8 to 1.1)	0.609
Week-10	− 6.5 (− 7.5 to − 5.5)^#^	− 6.6 (− 7.6 to − 5.5)^#^	0.1 (− 1.4 to 1.6)	0.894
Week-14	− 7.0 (− 7.9 to − 6.0)^#^	− 6.7 (− 7.8 to − 5.6)^#^	− 0.2 (− 1.7 to 1.2)	0.736
Week-18	− 7.6 (− 8.6 to − 6.6)^#^	− 7.2 (− 8.2 to − 6.1)^#^	− 0.5 (− 1.9 to 1.0)	0.541
Week-30	− 6.8 (− 7.8 to − 5.8)^#^	− 5.9 (− 7.0 to − 4.8)^#^	− 0.9 (− 2.3 to 0.6)	0.249
Week-42	− 7.5 (− 8.5 to − 6.5)^#^	− 6.7 (− 7.7 to − 5.6)^#^	− 0.8 (− 2.3 to 0.6)	0.265
*Secondary outcomes*				
Actiwatch, week-6				
SOL, min†	− 7.5 (− 9.8 to − 5.3)^#^	1.5 (− 0.9 to 3.9)	− 9.0 (− 12.3 to − 5.7)	< .001
WASO, min†	− 7.2 (− 12.1 to − 2.4)^#^	− 8.2 (− 13.5 to − 2.9)^#^	1.0 (− 6.2 to 8.2)	0.784
TST, min‡	2.7 (− 4.2 to 9.6)	2.3 (− 5.3 to 9.9)	0.4 (− 9.8 to 10.6)	0.943
SE, % ‡	2.8 (1.8 to 3.8)^#^	1.3 (0.3 to 2.4)	1.5 (0.0 to 2.9)	0.049
Sleep diary, week-6				
SOL, min†	− 11.4 (− 18.9 to − 3.9)^#^	− 3.3 (− 11.1 to 4.6)	− 8.1 (− 16.1 to − 0.2)	0.044
WASO, min†	− 9.2 (− 16.2 to 2.2)	− 13.5 (− 21.2 to − 5.8)^#^	4.3 (− 6.1 to 14.8)	0.413
TST, min‡	42.8 (32.2 to 53.3)^#^	13.5 (1.9 to 25.1)	29.2 (13.5 to 44.9)	< .001
SE, % ‡	8.6 (6.5 to 10.6)^#^	4.1 (1.8 to 6.4)^#^	4.4 (1.3 to 7.5)	0.005
PSQI†				
Week-3	− 2.3 (− 3.0 to − 1.5)^#^	− 2.1 (− 2.9 to − 1.4)^#^	− 0.1 (− 1.2 to 0.9)	0.808
Week-6	− 3.4 (− 4.2 to − 2.7)^#^	− 3.3 (− 4.2 to − 2.5)^#^	− 0.1 (− 1.2 to 1.0)	0.894
Week-10	− 4.1 (− 4.8 to − 3.3)^#^	− 3.9 (− 4.7 to − 3.1)^#^	− 0.2 (− 1.3 to 0.9)	0.742
Week-14	− 4.6 (− 5.3 to − 3.8)^#^	− 3.7 (− 4.5 to − 2.9)^#^	− 0.9 (− 2.0 to 0.2)	0.123
Week-18	− 5.0 (− 5.7 to − 4.2)^#^	− 4.5 (− 5.3 to − 3.7)^#^	− 0.5 (− 1.6 to 0.7)	0.408
Week-30	− 4.9 (− 5.6 to − 4.1)^#^	− 4.0 (− 4.9 to − 3.2)^#^	− 0.8 (− 2.0 to 0.3)	0.148
Week-42	− 5.2 (− 6.0 to − 4.4)^#^	− 4.2 (− 5.0 to − 3.3)^#^	− 1.0 (− 2.1 to 0.1)	0.084
HADS-Anxiety†				
Week-3	− 1.0 (− 1.6 to − 0.4)^#^	− 0.5 (− 1.1 to 0.1)	− 0.5 (− 1.3 to 0.3)	0.250
Week-6	− 2.0 (− 2.5 to − 1.4)^#^	− 1.2 (− 1.9 to − 0.6)^#^	− 0.7 (− 1.6 to 0.1)	0.098
Week-10	− 2.1 (− 2.7 to − 1.5)^#^	− 1.0 (− 1.7 to − 0.4)^#^	− 1.1 (− 1.9 to − 0.2)	0.016
Week-14	− 1.9 (− 2.5 to − 1.3)^#^	− 1.3 (− 2.0 to − 0.7)^#^	− 0.6 (− 1.4 to 0.3)	0.213
Week-18	− 2.3 (− 2.9 to − 1.7)^#^	− 1.8 (− 2.4 to − 1.1)^#^	− 0.5 (− 1.4 to 0.4)	0.256
Week-30	− 2.1 (− 2.7 to − 1.5)^#^	− 1.8 (− 2.5 to − 1.2)^#^	− 0.2 (− 1.1 to 0.6)	0.593
Week-42	− 2.6 (− 3.2 to − 2.0)^#^	− 1.0 (− 1.7 to − 0.4)^#^	− 1.6 (− 2.4 to − 0.7)	0.001
HADS-Depression†				
Week-3	− 0.7 (− 1.4 to − 0.1)^#^	− 0.8 (− 1.5 to − 0.1)^#^	− 0.1 (− 0.9 to 1.0)	0.889
Week-6	− 1.3 (− 1.9 to − 0.6)^#^	− 1.5 (− 2.2 to − 0.8)^#^	0.2 (− 0.7 to 1.2)	0.613
Week-10	− 2.0 (− 2.6 to − 1.3)^#^	− 1.5 (− 2.2 to − 0.8)^#^	− 0.5 (− 1.4 to 0.5)	0.354
Week-14	− 2.1 (− 2.8 to − 1.5)^#^	− 1.7 (− 2.4 to − 1.0)^#^	− 0.4 (− 1.4 to 0.5)	0.382
Week-18	− 2.2 (− 2.9 to − 1.6)^#^	− 2.2 (− 2.9 to − 1.5)^#^	− 0.0 (− 1.0 to 0.9)	0.932
Week-30	− 2.1 (− 2.8 to − 1.5)^#^	− 1.5 (− 2.3 to − 0.8)^#^	− 0.6 (− 1.6 to 0.4)	0.227
Week-42	− 3.2 (− 3.9 to − 2.5)^#^	− 1.3 (− 2.0 to − 0.6)^#^	− 1.9 (− 2.9 to − 0.9)	< .001
BFI†				
Week-3	− 1.0 (− 1.5 to − 0.6)^#^	− 1.1 (− 1.5 to − 0.6)^#^	0.0 (− 0.6 to 0.7)	0.917
Week-6	− 1.7 (− 2.2 to − 1.3)#	− 1.7 (− 2.2 to − 1.2)^#^	− 0.0 (− 0.7 to 0.6)	0.907
Week-10	− 2.1 (− 2.6 to − 1.7)^#^	− 1.6 (− 2.1 to − 1.1)^#^	− 0.5 (− 1.2 to 0.1)	0.117
Week-14	− 2.5 (− 2.9 to − 2.0)^#^	− 2.1 (− 2.6 to − 1.6)^#^	− 0.4 (− 1.0 to 0.3)	0.261
Week-18	− 2.6 (− 3.1 to − 2.2)^#^	− 2.2 (− 2.7 to − 1.7)^#^	− 0.4 (− 1.1 to 0.2)	0.189
Week-30	− 2.4 (− 2.9 to − 2.0)^#^	− 1.9 (− 2.4 to − 1.4)^#^	− 0.5 (− 1.2 to 0.1)	0.110
Week-42	− 2.7 (− 3.2 to − 2.2)^#^	− 2.1 (− 2.6 to − 1.6)^#^	− 0.5 (− 1.2 to 0.2)	0.134
BPI-SF pain severity†				
Week-3	− 0.7 (− 1.2 to − 0.2)^#^	− 0.5 (− 1.0 to 0.0)^#^	0.2 (− 0.9 to 0.5)	0.578
Week-6	− 0.6 (− 1.1 to − 0.1)	− 0.6 (− 1.2 to − 0.1)^#^	0.0 (− 0.7 to 0.8)	0.914
Week-10	− 1.0 (− 1.5 to − 0.5)^#^	− 1.2 (− 1.7 to − 0.6)^#^	0.2 (− 0.6 to 0.9)	0.642
Week-14	− 1.0 (− 1.4 to − 0.5)^#^	− 1.0 (− 1.5 to − 0.5)^#^	0.0 (− 0.7 to 0.8)	0.917
Week-18	− 0.7 (− 1.2 to − 0.2)	− 1.2 (− 1.7 to − 0.6)^#^	0.4 (− 0.3 to 1.2)	0.232
Week-30	− 1.0 (− 1.5 to − 0.5)^#^	− 1.2 (− 1.8 to − 0.7)^#^	0.3 (− 0.5 to 1.0)	0.470
Week-42	− 1.1 (− 1.6 to − 0.6)^#^	− 0.7 (− 1.2 to − 0.2)^#^	0.4 (− 1.1 to 0.4)	0.285
BPI-SF pain interference†				
Week-3	− 0.8 (− 1.3 to − 0.3)^#^	− 0.5 (− 1.0 to 0.0)^#^	− 0.3 (− 1.0 to 0.4)	0.375
Week-6	− 0.9 (− 1.4 to − 0.4)	− 0.8 (− 1.3 to − 0.3)^#^	− 0.1 (− 0.8 to 0.6)	0.817
Week-10	− 1.2 (− 1.7 to − 0.7)^#^	− 1.0 (− 1.5 to − 0.5)^#^	− 0.2 (− 0.9 to 0.5)	0.528
Week-14	− 1.5 (− 2.0 to − 1.0)^#^	− 1.3 (− 1.8 to − 0.8)^#^	− 0.2 (− 0.9 to 0.5)	0.530
Week-18	− 1.2 (− 1.7 to − 0.7)^#^	− 1.5 (− 2.1 to − 1.0)^#^	0.4 (− 0.4 to 1.1)	0.331
Week-30	− 1.2 (− 1.7 to − 0.7)^#^	− 1.6 (− 2.1 to − 1.1)^#^	0.4 (− 0.3 to 1.1)	0.300
Week-42	− 1.6 (− 2.1 to − 1.1)^#^	− 1.2 (− 1.7 to − 0.6)^#^	− 0.5 (− 1.2 to 0.3)	0.210
FACT-B‡				
Week-3	6.3 (3.3 to 9.4)^#^	6.8 (3.6 to 10.0)^#^	− 0.4 (− 4.9 to 4.0)	0.842
Week-6	10.6 (7.5 to 13.7)^#^	9.8 (6.5 to 13.1)^#^	0.8 (− 3.8 to 5.3)	0.742
Week-10	14.2 (11.1 to 17.3)^#^	10.5 (7.1 to 13.9)^#^	3.7 (− 0.9 to 8.3)	0.118
Week-14	14.7 (11.6 to 17.9)^#^	9.4 (6.0 to 12.8)^#^	5.3 (0.7 to 9.9)	0.024
Week-18	15.8 (12.6 to 18.9)^#^	12.3 (8.9 to 15.7)^#^	3.5 (− 1.2 to 8.1)	0.141
Week-30	14.4 (11.3 to 17.6)^#^	12.6 (9.2 to 16.0)^#^	1.8 (− 2.8 to 6.5)	0.441
Week-42	19.1 (15.9 to 22.3)^#^	10.1 (6.6 to 13.5)^#^	9.0 (4.3 to 13.7)	< .001

Data are presented as mean (95% confidence interval)

*BFI* Brief fatigue inventory; *BPI-SF* Brief pain inventory-short form; *ISI* Insomnia severity index; *PSQI* Pittsburgh sleep quality index; *SOL* Sleep-onset latency; *WASO* Wake after sleep onset; *TST* Total sleep time; *SE* Sleep efficiency; *HADS* Hospital anxiety and depression scale; *FACT-B* Functional assessment of cancer therapy-breast cancer

†A greater negative value represents improvement in symptoms

‡A greater positive value represents improvement in symptoms

^#^*P* < 0.05, calculated using a mixed-effects model with baseline adjustment to illustrate pre- and post-treatment within-group differences

^a^*P* value was calculated using a mixed-effects model with baseline adjustment to illustrate between-group differences

### Secondary outcomes

Secondary outcomes are summarized in Table 3. Within-group comparisons revealed that both groups had significant improvements from baseline on sleep quality, wake time after sleep onset, anxiety, depression, fatigue, pain, and quality of life (all *Ps* < 0.05). At 6 weeks, the active acupuncture was more effective than sham control in shortening sleep onset latency recorded with Actiwatch (*P* < 0.001) and diary (*P* = 0.044), increasing diary-recorded total sleep time (*P* < 0.001), Actiwatch (*P* = 0.049) and diary-measured (*P* = 0.005) sleep efficiency. Changes in PSQI did not differ between two groups over the whole study. The active acupuncture group had significantly greater effects than the sham control in reducing severity of HADS-measured anxiety at 10 weeks (*P* = 0.016) and both anxiety and depression at 42 weeks (*P* ≤ 0.001), and improving FACT-B-measured quality of life at 14 (*P* = 0.024) and 42 weeks (*P* < 0.001).

### Adverse events

Treatment-related AEs were mild (Additional file 1: eTable S5). Two non-treatment-related serious AEs (pneumonia) were reported in the active acupuncture group. The most common AE observed in the active acupuncture group was bruising [13.0% (6/69)]. There were 4 sham acupuncture-treated participants who reported auricular skin allergic reaction. No participants discontinued treatments due to AEs.

### Credibility for treatment and blinding design

Credibility score for treatment was not significantly different between groups (Additional file 1: eTable S6). James’ blinding index was 0.72 (95% CI 0.65–0.78; *P* = 1.000), and Bang’s blinding index was 0.13 (− 0.01–0.27; *P* = 0.038) for the active acupuncture and 0.09 (− 0.09–0.27; *P* = 0.150) for the sham control, indicating high successiveness of blinding (Additional file 1: eTables S7A and S7B).

## Discussion

The main purpose of this trial was to determine whether the active acupuncture regimen could produce superior efficacy over the sham control in improving chemotherapy-associated insomnia in breast cancer patients. Following 12 sessions of treatment, participants on the active acupuncture regimen showed significantly greater improvements than sham control in sleep onset latency, total sleep time and sleep efficiency, which were examined daily with the objective assessment Actiwatch and subjective assessment diary. Likewise, it is evidenced that active acupuncture was markedly associated with improvements in objective sleep parameters, including increases in total sleep time and sleep efficiency [60]. The present study showed that two groups did not differ in changes in ISI and PSQI scores, which were measured biweekly and monthly, respectively. The reasons for this discrepancy are twofold: firstly, the short-term efficacy of acupuncture in alleviating insomnia may be more apparent than long-term efficacy. Previous studies have demonstrated robust short-term effects of acupuncture for insomnia in breast cancer survivors and adult women [61, 62]. Secondly, daily-based sleep parameters from Actiwatch/diary seemed to be more sensitive in detecting improvements of insomnia than those assessed biweekly/monthly.

Participants on the active acupuncture regimen achieved better treatment outcomes than those on the sham control in reducing comorbid anxiety and depression and improving quality of life during treatment and follow-up. Similar results have been observed in recent trial that demonstrated the superior efficacy of active acupuncture regimen in improving chemotherapy-induced cognitive decline, distress caused psychological and somatic symptoms and social functions in breast cancer patients [29]. It is also consistent with numerous studies that confirm the effectiveness of acupuncture for anxiety and depressive disorders [63, 64]. Additionally, this study displayed that the active acupuncture group had a much lower discontinuation rate compared to the sham control. One common reason for discontinuation was lack of perceived efficacy. The current results affirm the notion that active acupuncture regimen is not only effective in improving insomnia and psychiatric disorders occurred during and post-chemotherapy in breast cancer patients, but such superior efficacy also could be well sustained over a follow-up period.

Moreover, in a follow-up period of 18–20 weeks, a much greater proportion of the participants in the active acupuncture group stopped taking sleeping medications than the sham control group. Our previous study, nonetheless, displayed no difference in cessation rate in long-term benzodiazepine users between electroacupuncture and non-invasive placebo control [65]. This seemed mainly due to the fact that occurrence of insomnia in this study was directly associated with chemotherapy, whereas insomnia in previous study was comorbid with psychiatric disorders [65]. It is therefore suggested that the active acupuncture regimen could serve as a tapering approach to reduce and even replace the use of sleeping medications in breast cancer patients.

Similar to previous studies [63, 66], this study presented that acupuncture had a high safety profile with well tolerability. There were only few mild adverse events reported from both groups and no participants discontinued treatments due to adverse events. In this study, while acupuncture therapy earned high trustworthiness from most participants, there was no difference between groups in credibility scores. The primary outcome was not deviated under adjustment for baseline expectancy, indicating that treatment outcomes were not confounded with participants’ expectancy.

Several possible mechanisms have been proposed to understand the effect of acupuncture for cancer-related insomnia. First, acupuncture can regulate cerebral neurotransmitters associated with sleep regulation, such as gamma aminobutyric acid, 5-hydroxytryptamine, dopamine, noradrenaline, acetylcholine, histamine and orexin [67, 68]. Second, acupuncture can reduce heart rate variability, blood pressure, and sympathetic nerve activity, which are frequently dysregulated in individuals with insomnia [69]. Acupuncture may counterbalance the over-excited sympathetic system, thus relieving insomnia symptoms. Third, the anti-inflammation of acupuncture may contribute to its effect for chemotherapy-associated insomnia [70]. Finally, acupuncture relieves physical (i.e., fatigue, pain, hot flushes, neurotoxicity) and psychological (i.e., depression, anxiety) comorbidities related to cancer and cancer treatment, and hence to improve sleep quality [71]. More research to investigate the mechanisms of acupuncture for cancer-related insomnia are warranted.

The efficacy of treatments might be affected by the precipitating or perpetuating factors of chemotherapy-associated insomnia [7, 8]. Acupuncture has been reported to generate an 8.3-point reduction in ISI score among cancer survivors who have completed active treatments [33], and about 6-point reduction among breast cancer patients undergo or post-chemotherapy in our present and previous trial [23]. Similarly, behavioral therapy produced a 10.9-point reduction in ISI score among cancer survivors [33], and around 6-point reduction in patients with breast cancer undergoing chemotherapy [72]. Although variations in treatment procedures and participant characteristics might partially explain the differences in efficacy of acupuncture or behavioral therapy among population with different cancer trajectory phases, the multiple physical and mental challenges faced by patients undergoing chemotherapy must be taken into account when comparing the results of these studies.

The sham control produced greater improvement in ISI score after 6-week of intervention than other sham-controlled acupuncture trials [21, 58], consequently led to the non-significant between-group difference and smaller effect size. The heterogeneous effects of sham acupuncture in different studies were associated with multiple factors. Firstly, the greater effect of sham acupuncture in the present study might be associated with longer treatment duration, more treatment sessions, and spontaneous relief of chemotherapy-related symptoms following the completion of chemotherapy. Secondly, 70% of participants had prior acupuncture experience, indicating participants had high expectancy in the effectiveness of treatment, thereby might overoptimize treatment responses. High expectancy might be related to high response rate in sham control which makes it more difficult to detect additional “specific” effects of active acupuncture over placebo [73]. Therefore, the tentatively non-inferiority effect of sham acupuncture seen in this study should be treated with caution.

One should consider the following limitations of this study. Firstly, although Streitberger’s retractable placebo needles have been widely used to differentiate specific effects of inserted acupuncture from non-inserted needles, it still could produce widespread modulatory effects at multiple levels of central nervous system by exciting mechanoreceptors underneath skin via pressure from non-inserted needles [28]. Numerous neuroimaging studies have demonstrated that there were no differences between “real” and “sham” acupuncture in neuromodulation of functional brain networks [28, 74]. Caution should be exercised in interpreting the tentatively non-inferiority effect of the sham acupuncture in this study. Recently, we have introduced minimum acupuncture stimulation as control, in which acupoints used are unrelated the treated condition, number of acupoints used and stimulation intensity are kept to a minimum at which patients were still aware of receiving active acupuncture [29, 75]. Secondly, the active acupuncture regimen is a combination of needling into body acupoints and acupressure on auricular points. It is unclear whether beneficial effects of such combination were generated from two individual acupuncture modes in a synergistic manner. Finally, only Actiwatch used as objective measure and discrepancies between Actiwatch and sleep diary were observed. Additional objective measures, e.g., polysomnography, should be considered reciprocally to validate the results from Actiwatch and clinical instruments. It also could help gain a better insight into brain mechanisms of the therapeutic effects of acupuncture for insomnia.

## Conclusion

The active acupuncture regimen produced better outcomes than the sham control in improving sleep onset latency, total sleep time and sleep efficiency in breast cancer women with chemotherapy-associated insomnia, although there was no significant difference in reducing insomnia severity. Active acupuncture had significantly greater effects in improving anxiety, depression and quality of life. Participants of the active acupuncture group showed a markedly higher cessation rate of sleeping medications. Active acupuncture regimen could be considered as an effective option for chemotherapy-associated insomnia and a tapering approach to reduce and even replace use of sleeping medications in breast cancer patients.

## Supplementary Information

**Additional file 1: eTable S1A.** Location and traditional Chinese medicine (TCM)-based therapeutic effects of the acupoints used in the trial. **eTable S1B.** Recommendation of additional acupoints based on common comorbid symptoms. **eTable S1C.** Detailed treatment procedures of active and sham acupuncture regimen. **eTable S2A.** Cessation rate, dose and weekly frequency of use of sleeping medications. **eTable S2B.** Characteristics of participants use sedatives, hypnotics, anxiolytics. **eTable S3.** Tipping-point sensitivity analysis for shift ISI score. **eTable S4.** Expectancy for treatment outcomes between groups. **eTable S5.** Adverse events related to treatment. **eTable S6.** Credibility toward treatment between the two groups. **eTable S7A.** Assessment of successiveness of blinding. **eTable S7B.** Results of blinding assessment.

## Data Availability

The data underlying this article will be shared on reasonable request addressed to zhangzj@hku.hk.

## Abbreviations

AE: Adverse event
AC: Adriamycin and cyclophosphamide
AC/EC: Adriamycin and cyclophosphamide, or epirubicin and cyclophosphamide
AES: Acupuncture expectancy scale
BFI: Brief fatigue inventory
BPI-SF: Brief pain inventory-short form
CMPs: Chinese medicine practitioners
DSMB: Data and safety monitoring board
FAC: Fluorouracil, adriamycin, and cyclophosphamide
FACT-B: Functional assessment of cancer therapy-breast cancer
FEC + T: Fluorouracil, epirubicin and cyclophosphamide, plus taxotere
HADS: Hospital anxiety and depression scale
HK: Hong Kong
HKU: University of Hong Kong
ISI: Insomnia severity index
MAR: Missing-at-random
SE: Sleep efficiency
SOL: Sleep onset latency
T/P: Taxotere or paclitaxel
TAC: Taxotere, adriamycin, and cyclophosphamide
TC: Taxotere and cyclophosphamide
TST: Total sleep time
WASO: Wake after sleep onset
