# Supplementary material for: Acupuncture for chemotherapy-associated insomnia in breast cancer patients: an assessor-participant blinded, randomized, sham-controlled trial
Source: Breast Cancer Res. 2023 Apr 26;25:49. doi: 10.1186/s13058-023-01645-0 (PMC10134666; doi:10.1186/s13058-023-01645-0)
Supplement: Supplementary file 1 — Additional file 1: eTable S1A. Location and traditional Chinese medicine (TCM)-based therapeutic effects of the acupoints used in the trial. eTable S1B. Recommendation of additional acupoints based on common comorbid symptoms. eTable S1C. Detailed treatment procedures of active and sham acupuncture regimen. eTable S2A. Cessation rate, dose and weekly frequency of use of sleeping medications. eTable S2B. Characteristics of participants use sedatives, hypnotics, anxiolytics. eTable S3. Tipping-point sensitivity analysis for shift ISI score. eTable S4. Expectancy for treatment outcomes between groups. eTable S5. Adverse events related to treatment. eTable S6. Credibility toward treatment between the two groups. eTable S7A. Assessment of successiveness of blinding. eTable S7B. Results of blinding assessment. [file 13058_2023_1645_MOESM1_ESM.docx]

**Supplement**

of

**Effectiveness of acupuncture for chemotherapy-associated insomnia in breast cancer patients: An assessor-participant blinded, randomized, sham-controlled trial**

Jialing Zhang; Zongshi Qin; Tsz Him So; Tien Yee Chang; Sichang Yang; Haiyong Chen; Wing Fai Yeung; Ka Fai Chung; Pui Yan Chan; Yong Huang; Shifen Xu; Chun Yuan Chiang; Lixing Lao; Zhang-Jin Zhang

**eTable S1A.** Location and traditional Chinese medicine (TCM)-based therapeutic effects of the acupoints used in the trial

**eTable S1B.** Recommendation of additional acupoints based on common comorbid symptoms

**eTable S1C.** Detailed treatment procedures of active and sham acupuncture regimen

**eTable S2A.** Cessation rate, dose and weekly frequency of use of sleeping medications

**eTable S2B.** Characteristics of participants use sedatives, hypnotics, anxiolytics

**eTable S3.** Tipping-point sensitivity analysis for shift ISI score

**eTable S4.** Expectancy for treatment outcomes between groups

**eTable S5.** Adverse events related to treatment

**eTable S6.** Credibility towards treatment between the two groups

**eTable S7A.** Assessment of successiveness of blinding

**eTable S7B.** Results of blinding assessment

This supplemental material has been provided by the authors to give readers additional information about their work.

## **eTable S1A.** Location and traditional Chinese medicine (TCM)-based therapeutic effects of the acupoints used in the trial

| **Acupoint** | **Location** | **TCM-based therapeutic effects** |
| --- | --- | --- |
| **Fixed Acupoints**  *Used on all participants* | | |
| **GV24 (Shenting)** | On the head at the anterior median line, 0.5 *cun* superior to the anterior hairline. | Subdues *Yang* and calms the spirit, has a wide disease spectrum including insomnia, depression, mania and other mental disorders. |
| **GV20**  **(Baihui)** | On the head at the anterior median line, 5 *cun* superior to the anterior hairline. | Raises *Yang*, regulates the five *zang*’s activities, strengthens brain, and relieves mental stress, has remarkable effects on mental disorders, in particular insomnia, depression, and anxiety. |
| **EX-HN1 (Sishencong)** | At the vertex of the scalp; four points, 1 *cun* respectively anterior, posterior, and lateral to GV20 (Baihui). | Tranquilize and calm the mind,  help in suppressing sympathetic activities, and are commonly used for insomnia, pain, fatigue and depression. |
| **PC6**  **(Neiguan, bilateral)** | On the medial aspect of the forearm between the palmaris longus and flexor carpi radialis tendons, 2 *cun* proximal to the palmar wrist crease. | Calm the mind and heart, calm and relieves pain, strongly inhibit sympathetic tone, with reduction of cardiac frequency, systolic blood pressure and an important reduction of the amplitude of the sympathetic electrical response. Commonly used for insomnia, fatigue, pain and depression. |
| **SP6 (Sanyinjiao, bilateral)** | On the tibial aspect of the leg posterior to the medial border of the tibia, 3 *cun* superior to the prominence of the medial malleolus. | Tonify *Yin* and improve vitality and immunity. Nourish the three foot meridians, including spleen, kidney and liver. Widely used for problems of endocrine and immune system, including insomnia, fatigue and pain. |
| **KI3**  **(Taixi, bilateral)** | On the posteromedial aspect of the ankle in the depression between the prominence of the medial malleolus and the calcaneal tendon. | Replenish kidney *Yin*. Widely used for regulating endocrine system. Suitable for relieving fatigue. |
| **Additional Acupoints**  *4 additional acupoints are selected on the basis of patients’ comorbid symptoms* | | |
| **EX-HN3 (Yintang)** | At the midpoint between the medial ends of the eyebrows. | Quiets the spirit and clears the mind. Widely used in treating insomnia, headache, vertigo, forgetfulness, epilepsy, infantile convulsion and nose problems. |
| **EX-HN22 (Anmian)** | Behind the ear, midway between GB-20 (Fengchi) and SJ-17 (Yifeng), close but slightly posterior and inferior to GB-12 (Wangu). | Beneficial in improving sleeping quality and is commonly used for insomnia. |
| **LI4**  **(Hegu)** | Dorsum of hand, at the level of the midpoint of the second metacarpal bone, between first and second metacarpal bones. | Expels *Wind* and releases the exterior, tonifies *Qi* and strengthens immunity. Used to manage various type of psychogenic tense and pain. |
| **HT7 (Shenmen)** | On the posteromedial aspect of the wrist radial to the flexor carpi ulnaris tendon at the palmar wrist crease. | Chiefly for calming the mind and tranquilizing the spirit. Used for a variety of psychogenic conditions, including insomnia, anxiety and depression. |
| **CV4 (Guanyuan)** | Abdomen, 3 *cun* under the navel. | Tonifies *Qi* and supplements *Yang*. Modulates the limbic-medial prefrontal network related to cognitive function. Beneficial for constipation, retention of urination, frequent nocturia and indigestion. |
| **ST25 (Tianshu)** | Abdomen, 2 *cun* away from the navel. | Regulates *Qi*, regulates the center and invigorates the spleen. Beneficial for bipolarism, epilepsy, insomnia, dizziness and headache. |
| **ST36**  **(Zusanli)** | Antero-lateral leg, 1 middle finger breadth next to the anterior crest of tibia, 3 *cun* under the depression lateral to the patellar ligament. | Invigorates spleen, harmonizes stomach, reinforces healthy and original *Qi*. Widely used for fatigue management and pain alleviation. Has broad therapeutic effects, from gastro, intestinal and endocrinal diseases to neuropsychiatric disorders. Evokes the robust response in limbic-paralimbic-neocortical network involved in autonomic, pain, mood and cognitive function. |
| **KI6**  **(Zhaohai)** | On the medial aspect of the foot in the depression inferior to the medial malleolus 1 *cun* inferior to the prominence of the medial malleolus. | Confluence point of the *Yin Qiao Mai*. Nourishes *Yin* and calms the mind. Commonly used for treating insomnia. |
| **LR3 (Taichong)** | Dorsum of foot, within the depression between first and second metatarsal bones. | Quiets the spirit and stabilizes the mind. Great points for psychological disorders, depression and anxiety. Can be used for various kinds of pain. Often used to control fire-syndrome caused by stagnation of liver-*Qi* or fire hyperactivity because of *Yin* deficiency. |
| **Auricular Points**  *Auricular points used in acupuncture group include Shenmen (TF4), Sympathetic (AH6a), and Heart (CO15).*  *Auricular points used in sham acupuncture group include* *3 Helix points (HX7, HX8, and HX9).* | | |
| **Shenmen (TF4)** | The supper part of the inferior 1/3 of the triangular fossa. | Effectively improves the sleep quality and thus is commonly used for treating insomnia, helps in increasing oxygen uptake and decreasing heart rate. |
| **Sympathetic (AH6a)** | Anterior to AH6, at the junction of the superior border of the inferior antihelix crus and the medial border of helix. | Helps in functional disorders of autonomic nervous system, such as palpitation, hot flashes, and insomnia. |
| **Heart (CO15)** | The center of the depression of the cavum concha at CO15. | Helps in calming the mind and spirit. Beneficial in improving the sleep quality |
| **Helix points at HX7** | On the helix, the midpoint at HX7. | Remote from the inner ear area and their therapeutic effects are not indicated for the treatment of insomnia according to TCM theory. |
| **Helix points at HX8** | On the helix, the midpoint at HX8. |  |
| **Helix points at HX9** | On the helix, the midpoint at HX9. |  |

## **eTable S1B.** Detailed treatment procedures of active and sham acupuncture regimen

| **Acupuncture regimen** | **Detailed procedures** |
| --- | --- |
| **Active acupuncture regimen** |  |
| *Electroacupuncture (EA)* | - Acupoints: 6 fixed acupoints plus 4 additional acupoints were used. - Procedures: Following cleansing of the skin of acupoints with alcohol swab, a sterilized disposable filiform needle (0.2 mm × 25-40 mm in a guiding tube) was inserted to a depth of 0.5-1 cm. Manual manipulation was then performed to elicit needling (De-Qi) sensation. Electrical stimulation with continuous wave at 2-5 Hz was conducted by connecting 4 pairs of electrodes (AWQ-104L, Electro Therapeutic Devices Inc.) to Baihui (+) and Shenting (–), left and right Sishencong (L+, R–), and other two pairs on acupoints chosen based on patients’ individual symptoms. - Duration: The stimulation lasted for 25 minutes. |
| *Auricular acupressure* | - Points: Heart, Shenmen, Sympathetic. - Procedures: Following the completion of EA, auricular acupressure was conducted by embedding black, hard Vaccaria seeds (Wang Bu Liu Xing, 1-2 mm in diameter) on the surface of bilateral 3 auricular points. Participants were instructed to gently press 1 min each point for three times every day. - Duration: Re-embedded periodically at an interval of 2-3 days. |
| **Sham acupuncture regimen** |  |
| *Sham EA* | - Points: 10 sham points were used. Sham points that are defined as the points located at 1-2 cm adjacent to meridian-based acupoints. - Procedures: Streitberger’s non-invasive, retractable blunt tipped needles were compressed via guiding tubes onto the skin of sham points, where the needles were actually not penetrated, but participants felt needle insertion. Then the needles were held using surgical tape without manual manipulation and connected to an electric stimulator in which electrical current was not delivered. - Duration: 25 minutes. |
| *Sham auricular acupressure* | - Points: HX7, HX8, HX9. - Procedures: Following the completion of sham EA, soft stem piths of Medulla Junci (Deng Xin Cao), a Chinese medicinal material which were cut into 1-2 mm in length and dyed in black were used to mimic auricular acupressure by embedding onto the surface of three sham auricular points. Participants were instructed not to press these soft piths. - Duration: Re-embedded periodically at an interval of 2-3 days. |

## **eTable S2A.** Cessation rate, dose and weekly frequency of use of sleeping medications

| **Sedatives, hypnotics, anxiolytics use** | **Active acupuncture**  **(n = 26)** | **Sham acupuncture**  **(n = 19)** | ***P* value** * |
| --- | --- | --- | --- |
| **Cessation rate, %** |  |  |  |
| Week 6 to 8 | 9 (37.5) | 3 (20.0) | 0.305 |
| Week 18 to 20 | 13 (56.5) | 2 (14.3) | 0.011 |
| Week 40 to 42 | 13 (56.5) | 3 (23.1) | 0.083 |
| **Equivalent dose of usage in diazepam, mg/d** |  |  |  |
| Baseline | 3.29 ± 2.31 | 2.88 ± 3.54 | 0.638 |
| Week 6 to 8 | 1.17 ± 1.58 | 1.78 ± 1.76 | 0.271 |
| Week 18 to 20 | 1.12 ± 1.86 | 1.23 ± 1.25 | 0.843 |
| Week 40 to 42 | 1.23 ± 2.04 | 1.54 ± 1.54 | 0.635 |
| **Weekly frequency of use, dose/week** |  |  |  |
| Baseline | 4.66 ± 2.41 | 4.32 ± 2.82 | 0.659 |
| Week 6 to 8 | 2.60 ± 3.23 | 3.95 ± 3.15 | 0.210 |
| Week 18 to 20 | 2.22 ± 3.24 | 3.48 ± 3.31 | 0.261 |
| Week 40 to 42 | 2.22 ± 3.24 | 3.92 ± 3.22 | 0.138 |

Note: Data are presented as mean ± standard deviation or number (%).

* Comparison between groups by $\boldsymbol{\chi}$***^2^*** or unpaired *t*-test.

## **eTable S2B**. Characteristics of participants use sedatives, hypnotics, anxiolytics (n=45)

| **Characteristic** | **Active acupuncture**  **(n = 26)** | **Sham acupuncture**  **(n = 19)** | ***P* value*** |
| --- | --- | --- | --- |
| **Age, years** | 49.5 ± 8.9 | 55.3 ± 10.9 | 0.056 |
| **Body mass index, kg/m^2^** | 22.4 ± 3.1 | 21.3 ± 2.6 | 0.232 |
| **Marital status** |  |  | 0.036 |
| Married or living with partner | 19 (73.1) | 8 (42.1) |  |
| Single, separated, divorced, or widowed | 7 (26.9) | 11 (57.9) |  |
| **Educational attainment** |  |  | 0.910 |
| Primary | 2 (7.7) | 2 (10.5) |  |
| Secondary or below | 10 (38.5) | 8 (42.1) |  |
| Post-secondary or above | 14 (53.8) | 9 (47.4) |  |
| **Household monthly income (HK$)** |  |  | 0.386 |
| ＜20,000 | 13 (50.0) | 11 (57.9) |  |
| 20,000-50,000 | 7 (26.9) | 2 (10.5) |  |
| ＞50,000 | 6 (23.1) | 6 (31.6) |  |
| **Occupation** |  |  | 0.666 |
| Professional and associate professional | 10 (38.5) | 5 (26.3) |  |
| Unskilled worker | 5 (19.2) | 3 (15.8) |  |
| Retired/unemployed/housework | 11 (42.3) | 11 (57.9) |  |
| **Menopausal status at entry** |  |  | 0.840 |
| Premenopausal | 1 (3.8) | 1 (5.3) |  |
| Perimenopausal | 5 (19.2) | 2 (10.5) |  |
| Postmenopausal | 20 (76.9) | 16 (84.2) |  |
| **Breast cancer stage** |  |  | 0.424 |
| I | 2 (7.7) | 4 (21.1) |  |
| II | 13 (50.0) | 11 (57.9) |  |
| III | 6 (23.1) | 2 (10.5) |  |
| IV | 5 (19.2) | 2 (10.5) |  |
| **Prior surgery** | 23 (88.5) | 17 (89.5) | 0.915 |
| **Prior radiotherapy** | 13 (50.0) | 10 (52.6) | 0.862 |
| **Prior hormonal therapy** | 11 (42.3) | 10 (52.6) | 0.493 |
| **Adjuvant Chemotherapy** | 19 (73.1) | 15 (78.9) | 0.856 |
| **Under or post chemotherapy at entry** |  |  | 0.734 |
| Under | 7 (26.9) | 6 (31.6) |  |
| Post | 19 (73.1) | 13 (68.4) |  |
| **Chemotherapy regimens** |  |  | 0.676 |
| AC/TC | 4 (15.4) | 6 (31.6) |  |
| TAC | 4 (15.4) | 2 (10.5) |  |
| AC/EC + T/P | 7 (26.9) | 2 (10.5) |  |
| FEC + T | 2 (7.7) | 1 (5.3) |  |
| Carboplatin-containing | 6 (23.1) | 6 (31.6) |  |
| Others | 3 (11.5) | 2 (10.5) |  |
| **Insomnia mean duration, months** ^a^ | 7.0 (3.0, 13.3) | 13.0 (5.0, 17.0) | 0.035 |
| **Sleep aids, prior 2 weeks** |  |  |  |
| Chinese herbal medicine | 6 (23.1) | 9 (47.4) | 0.088 |
| **Prior acupuncture** | 20 (76.9) | 15 (68.9) | 0.872 |
| **ISI** | 18.5 ± 4.2 | 17.3 ± 3.7 | 0.295 |
| **PSQI** | 15.7 ± 2.6 | 14.8 ± 3.2 | 0.185 |
| **Actiwatch** |  |  |  |
| SOL, min | 11.2 ± 12.5 | 15.9 ± 15.0 | 0.256 |
| WASO, min | 111.8 ± 37.8 | 126.9 ± 48.5 | 0.248 |
| TST, min | 383.3 ± 39.5 | 368.8 ± 54.1 | 0.305 |
| SE, % | 74.7 ± 6.5 | 70.9 ± 9.4 | 0.118 |
| **Sleep diary** |  |  |  |
| SOL, min | 60.3 ± 50.5 | 60.3 ± 44.5 | 0.997 |
| WASO, min | 51.1 ± 32.2 | 56.3 ± 64.6 | 0.726 |
| TST, min | 334.4 ± 92.2 | 313.1 ± 117.9 | 0.500 |
| SE, % | 64.1 ± 14.5 | 61.5 ± 21.3 | 0.622 |
| **HADS** |  |  |  |
| Anxiety | 11.0 ± 4.2 | 8.2 ± 2.9 | 0.016 |
| Depression | 9.9 ± 4.5 | 8.1 ± 4.5 | 0.206 |
| **BFI** | 6.2 ± 2.0 | 5.9 ± 1.7 | 0.580 |
| **BPI-SF** |  |  |  |
| Pain severity | 3.3 ± 3.0 | 4.5 ± 2.4 | 0.164 |
| Pain interference | 3.4 ± 3.2 | 4.4 ± 2.8 | 0.253 |
| **FACT-B** | 73.2 ± 19.7 | 80.2 ± 19.1 | 0.243 |
| **AES** | 15.3 ± 3.2 | 14.5 ± 2.9 | 0.372 |

Note: Data are presented as mean ± standard deviation, number (%), or median (IQR)

Abbreviations: AC/TC, Adriamycin and Cyclophosphamide, or Taxotere and Cyclophosphamide; AC/EC, Adriamycin and Cyclophosphamide, or Epirubicin and Cyclophosphamide; BFI, Brief Fatigue Inventory; BPI-SF, Brief Pain Inventory-Short Form; FEC + T, Fluorouracil, Epirubicin and Cyclophosphamide, plus Taxotere; ISI, Insomnia Severity Index; PSQI, Pittsburgh Sleep Quality Index; SOL, sleep onset latency; TAC, Taxotere, Adriamycin, and Cyclophosphamide; T/P, Taxotere or Paclitaxel; WASO, wake after sleep onset; TST, total sleep time; SE, sleep efficiency; HADS, Hospital Anxiety and Depression Scale; FACT-B, Functional Assessment of Cancer Therapy-Breast Cancer; AES, Acupuncture Expectancy Scale.

^a^ Duration of insomnia was reported by the participant and verified by assessors before enrolment, and compared with Mann-Whitney U test.

* Comparison between acupuncture group and sham acupuncture group by $\boldsymbol{\chi}$***^2^*** or Fisher’s exact test, or unpaired *t*-test.

## **eTable S3.** Tipping-point sensitivity analysis for shift ISI score

| **No.** | **Shift parameter (ISI score)** | ***P-*value^*^** |
| --- | --- | --- |
| 1 | 7 | 0.9949 |
| 2 | 8 | 0.9731 |
| 3 | 9 | 0.9411 |
| 4 | 10 | 0.9217 |
| 5 | 11 | 0.8774 |
| 6 | 12 | 0.8545 |
| 7 | 13 | 0.8420 |
| 8 | 14 | 0.7845 |
| 9 | 15 | 0.7589 |
| 10 | 16 | 0.7443 |
| 11 | 17 | 0.7326 |
| 12 | 18 | 0.6967 |
| 13 | 19 | 0.6672 |
| 14 | 20 | 0.6548 |
| 15 | 21 | 0.6421 |
| 16 | 22 | 0.6217 |
| 17 | 23 | 0.5942 |
| 18 | 24 | 0.5723 |
| 19 | 25 | 0.5569 |
| 20 | 26 | 0.5418 |
| 21 | 27 | 0.5263 |
| 22 | 28 | 0.5116 |

Abbreviation: ISI, Insomnia Severity Index

* Between-group comparison at week 6.

## **eTable S4.** Expectancy for treatment outcomes between groups

| **AES total score** | **Active acupuncture**  **(n = 69)** | **Sham acupuncture**  **(n = 69)** | ***P* value** * |
| --- | --- | --- | --- |
| Week-0 | 14.8 (14.0 to 15.6) | 14.8 (14.0 to 15.6) | 0.990 |
| Week-3 | 12.9 (12.1 to 13.8) | 12.3 (11.5 to 13.2) | 0.296 |
| Week-6 | 13.4 (12.5 to 14.2) | 13.2 (12.3 to 14.1) | 0.750 |
| Week-10 | 13.9 (13.0 to 14.7) | 12.8 (11.9 to 13.7) | 0.102 |
| Week-14 | 13.4 (12.6 to 14.3) | 11.8 (10.9 to 12.7) | 0.009 |
| Week-18 | 13.5 (12.7 to 14.4) | 12.8 (11.8 to 13.7) | 0.214 |
| Week-30 | 12.9 (12.0 to 13.7) | 11.5 (10.6 to 12.4) | 0.031 |
| Week-42 | 13.3 (12.4 to 14.1) | 11.6 (10.7 to 12.5) | 0.008 |

Note: Data are presented as mean (95% Confidence Interval).

Abbreviations: AES, Acupuncture Expectancy Scale.

* *P* value was calculated using a mixed-effects model with baseline adjustment to illustrate between-group differences.

## **eTable S5.** Adverse events related to treatment

| **Adverse event** | **Active acupuncture (n = 69)** | **Sham acupuncture (n = 69)** |
| --- | --- | --- |
| Auricular skin allergic reaction | 3 (4.3%) | 4 (5.8%) |
| Hematoma around the site of needling | 9 (13.0%) | 0 (0.0%) |
| Localized pain | 1 (1.4%) | 2 (2.9%) |
| Nausea during acupuncture | 1 (1.4%) | 0 (0.0%) |
| Dizziness after acupuncture | 2 (2.9%) | 1 (1.4%) |
| Headache after acupuncture | 1 (1.4%) | 0 (0.0%) |

Note: Data are presented as frequency.

## **eTable S6.** Credibility towards treatment between the two groups

| **Credibility score** | **Active acupuncture**  **(n = 69)** | **Sham acupuncture**  **(n = 69)** | ***P* value** * |
| --- | --- | --- | --- |
| **After the 3^rd^ treatment** |  |  |  |
| **Q1:** How confident do you feel that this treatment can alleviate your complaint? | 4.7 ± 1.0 | 4.7 ± 1.0 | 0.908 |
| **Q2:** How confident would you be recommending this treatment you a friend who suffered from similar complaints? | 4.7 ± 1.0 | 4.7 ± 1.0 | 0.972 |
| **Q3:** How logical does this treatment seem to you? | 4.9 ± 0.9 | 4.8 ± 0.9 | 0.474 |
| **Q4:** How successful do you think this treatment would be in alleviating other complaints? | 4.6 ± 0.9 | 4.4 ± 1.0 | 0.264 |
| **Total** | 18.8 ± 3.5 | 18.5 ± 3.5 | 0.598 |
| **After the 15^th^ treatment** |  |  |  |
| **Q1** | 4.4 ± 1.2 | 4.3 ± 1.3 | 0.639 |
| **Q2** | 4.5 ± 1.2 | 4.3 ± 1.4 | 0.353 |
| **Q3** | 4.8 ± 1.0 | 4.6 ± 1.2 | 0.374 |
| **Q4** | 4.6 ± 1.0 | 4.4 ± 1.2 | 0.502 |
| **Total** | 18.3 ± 4.0 | 17.6 ± 4.9 | 0.431 |

Note: Data are presented as mean ± standard deviation.

* Comparison by unpaired *t*-test.

## **eTable S7A.** Assessment of successiveness of blinding

|  | **Active acupuncture** | **Sham acupuncture** |
| --- | --- | --- |
| **Group guessed after the 3^rd^ treatment** |  |  |
| 1. Acupuncture treatment | 18 (26.1) | 14 (21.5) |
| 2. Sham acupuncture treatment | 9 (13.0) | 20 (30.8) |
| 3. Uncertain | 42 (60.9) | 31 (47.7) |

Note: Data are presented as number (%).

## **eTable S7B.** Results of blinding assessment

| **Methods** | **Index** | ***P­*-value** | **95% Confidence interval** | **Conclusion** |
| --- | --- | --- | --- | --- |
| James | 0.72 | 1.000 | 0.65 to 0.78 | Blinded |
| Bang-Acupuncture/2×3 | 0.13 | 0.038 | -0.01 to 0.27 | Blinded |
| Bang-Sham/2×3 | 0.09 | 0.150 | -0.09 to 0.27 | Blinded |
